# Recent advances and challenges of repurposing nanoparticle-based drug delivery systems to enhance cancer immunotherapy

**DOI:** 10.7150/thno.38425

**Published:** 2019-10-16

**Authors:** Seungho Lim, Jooho Park, Man Kyu Shim, Wooram Um, Hong Yeol Yoon, Ju Hee Ryu, Dong-Kwon Lim, Kwangmeyung Kim

**Affiliations:** 1 Center for Theragnosis, Biomedical Research Institute, Korea Institute of Science and Technology (KIST), 5, Hwarangno 14-gil, Seongbuk-gu, Seoul, 02792, Republic of Korea; 2 KU-KIST Graduate School of Converging Science and Technology, Korea University, 145, Anam-ro, Seongbuk-gu, Seoul, 02841, Republic of Korea.

**Keywords:** Nanoparticles, drug delivery system, immunogenic cell death, adjuvants, cytokines, and cancer immunotherapy

## Abstract

Cancer immunotherapy is an attractive treatment option under clinical settings. However, the major challenges of immunotherapy include limited patient response, limited tumor specificity, immune-related adverse events, and immunosuppressive tumor microenvironment. Therefore, nanoparticle (NP)-based drug delivery has been used to not only increase the efficacy of immunotherapeutic agents, but it also significantly reduces the toxicity. In particular, NP-based drug delivery systems alter the pharmacokinetic (PK) profile of encapsulated or conjugated immunotherapeutic agents to targeted cancer cells or immune cells and facilitate the delivery of multiple therapeutic combinations to targeted cells using single NPs. Recently, advanced NP-based drug delivery systems were effectively utilized in cancer immunotherapy to reduce the toxic side effects and immune-related adverse events. Repurposing these NPs as delivery systems of immunotherapeutic agents may overcome the limitations of current cancer immunotherapy. In this review, we focus on recent advances in NP-based immunotherapeutic delivery systems, such as immunogenic cell death (ICD)-inducing drugs, cytokines and adjuvants for promising cancer immunotherapy. Finally, we discuss the challenges facing current NP-based drug delivery systems that need to be addressed for successful clinical application.

## 1. Introduction

Cancer immunotherapy is emerging as an attractive treatment option for patients diagnosed with cancer, resulting in dramatic clinical results. The remarkable success of monoclonal antibodies as immune checkpoint inhibitors in clinical settings, and their subsequent approval by the United States Food and Drug Administration (FDA) has changed the landscape of cancer treatment 1, 2. However, the benefit of most cancer immunotherapies has been limited to only a minority of patients and indications 3. To overcome this poor efficacy of cancer immunotherapy, several attempts have been made using combination therapies consisting of multiple immune checkpoint inhibitors in clinical settings. Based on preclinical and clinical studies, combination therapy usually enhances therapeutic efficacy in cancer treatment, but can also lead to immune-related adverse effects in addition to the high cost involved. In addition, many immune checkpoint inhibitors administered systemically have limited tumor specificity, which can cause off-target adverse effects due to immune reactions against normal tissues 4-6. The off-target adverse effects can range from relatively minor conditions such as skin redness or blisters, to severe adverse effects such as pneumonitis, colitis, and endocrinopathies 7, 8. Therefore, novel approaches are urgently required to increase the efficacy of cancer immunotherapy by minimizing off-target adverse effects.

Various NP-based drug delivery systems have been extensively developed as vehicles for targeted cancer delivery of anticancer agents, such as chemical drugs, small interfering RNAs, DNAs, cytokines, and antibodies for over 30 years 9-12. They have demonstrated tumor-specific delivery of anticancer agents, owing to the preferential accumulation of NPs at targeted tumors and enhanced permeability and retention (EPR) effect 13. In addition to EPR effect, NPs have been used to actively target drug delivery to the desired cancer cells via specific binding to target receptors overexpressed on the surface of cancer cells. Active targeting may be achieved by simply introducing targeting moieties (e.g., peptides, aptamers, antibodies) on the surface of NPs 14. This target-specific delivery of anticancer drugs based on EPR and active targeting has been shown to greatly enhance therapeutic efficacy, and successfully diminish undesirable off-target toxicity 15-17.

**Figure 1:**
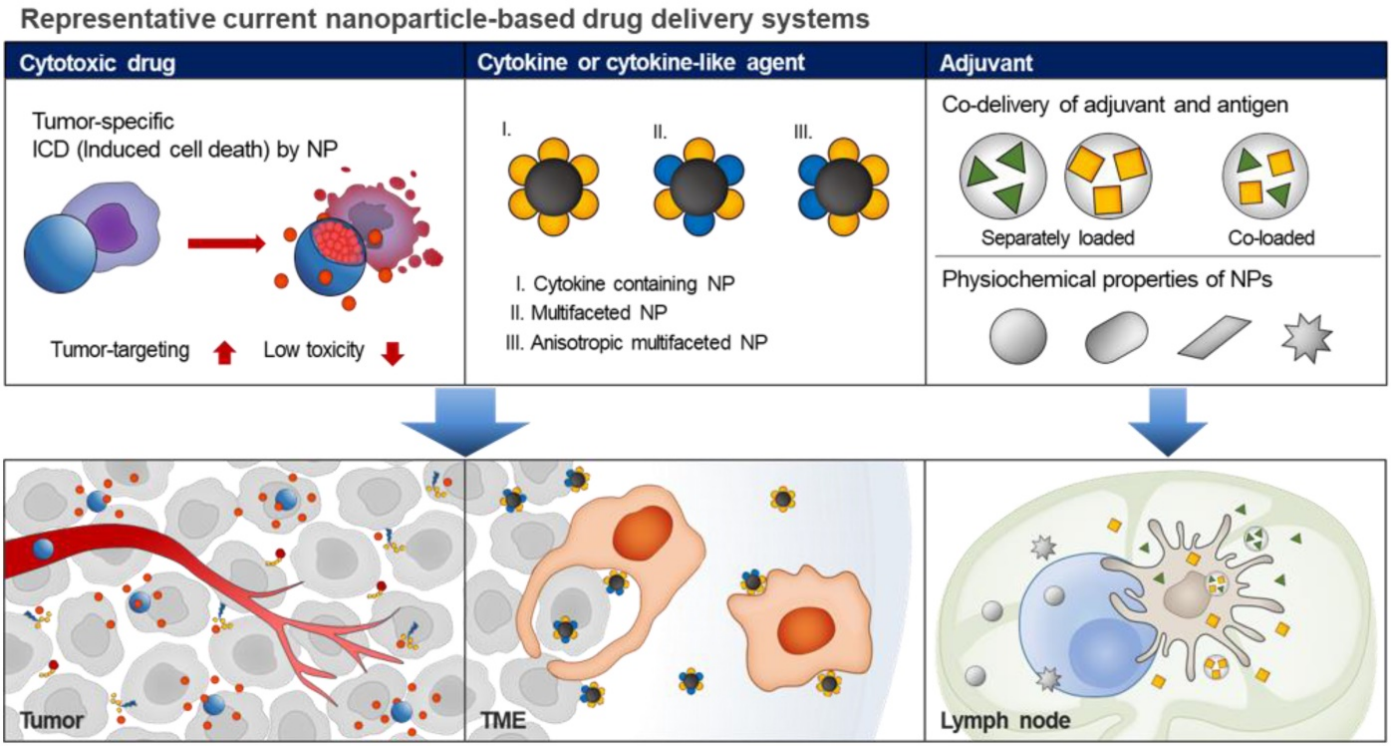
Schematic illustrations of current nanoparticle-based drug delivery systems for cancer immunotherapy. Based on the EPR effect and active targeting strategies, the current nanoparticle-based drug delivery systems can be used for targeted delivery of cytotoxic drugs to the tumor tissue, resulting in ICD induction. In addition, the nanoparticle-based cytokine delivery system for the delivery of multiple immunotherapeutic agents to target tumor cells or immune cells, results in immune responses. In the case of adjuvant delivery, the nanoparticle-based delivery systems with fine-tuned sizes, shapes, surface charges, and hydrophobicity can be used for the delivery of immunotherapeutics into tumor tissues or lymph nodes.

Repurposing these NP-based drug delivery systems for immunotherapeutic agents to target the immune system may overcome the lower efficacy, immune-related adverse events, and off-targeted toxicity of currently used agents (**Figure 1**). Notably, the use of NPs for immunotherapeutic delivery can offer the following benefits. First, NPs containing one or more immunotherapeutic agents can easily target the desired cancer or immune cells. The targeted delivery of NPs to cancer cells can be accomplished based on EPR effect and active targeting strategies such as tumor-targeting nanomedicine. Targeted delivery of NPs to immune cells or immune cell subsets can be further enhanced via chemical or physical modification of immune system-targeting ligands that specifically bind to overexpressed receptors on the surface of target cancer or immune cells 18-20. Several studies have shown that fine-tuning of sizes, shapes, surface charges, and hydrophobicity of NPs successfully improved the delivery of immunotherapeutic agents into tumor tissues or lymph nodes (LNs) 21, 22. Second, each NP can be designed to incorporate multiple immunotherapeutic agents for delivery to cancer targets cancer or immune cells simultaneously 23. Many studies have demonstrated that co-delivery of immunotherapeutic agents within a single cell enhanced the immune response and optimized the antigen processing and presentation. Finally, NPs containing immunotherapeutic agents show controlled drug release in response to complex tumor microenvironments (TME) (e.g., enzymes, hypoxia and acidic pH) or external stimuli (e.g., light, ultrasound, and electricity), which can greatly increase the efficiency of targeted drug delivery in cancer immunotherapy 24. Therefore, NP-based drug delivery systems can be used to enhance the efficacy of immunotherapeutic agents such as antibodies and cytokines in cancer immunotherapy.

In this review, we tried to discuss repurposing inherent properties (e.g. encapsulation of chemodrugs, target-specific delivery, controlling PK of drugs, etc.) of NP-based delivery systems to immunotherapeutics for improving therapeutic efficacy in cancer immunotherapy. In this point of view, recent studies which provided implications for overcoming current challenges in immunotherapeutics were selected and categorized into three types (Immunogenic cell death-inducing cytotoxic NPs, cytokines and cytokine-like immune modulator NPs, and adjuvants). Furthermore, we highlighted the potential of NP-based delivery systems that could overcome the current limitations of cancer immunotherapy. Finally, we discussed the expected challenges associated with repurposing such drug delivery systems for cancer immunotherapy, including low levels of tumor targeting, complex mechanism of therapeutic efficiency and limited commercial success.

## 2. Advanced NP-based drug delivery systems of immunogenic cell death (ICD)-inducing cytotoxic drugs

**Figure 2:**
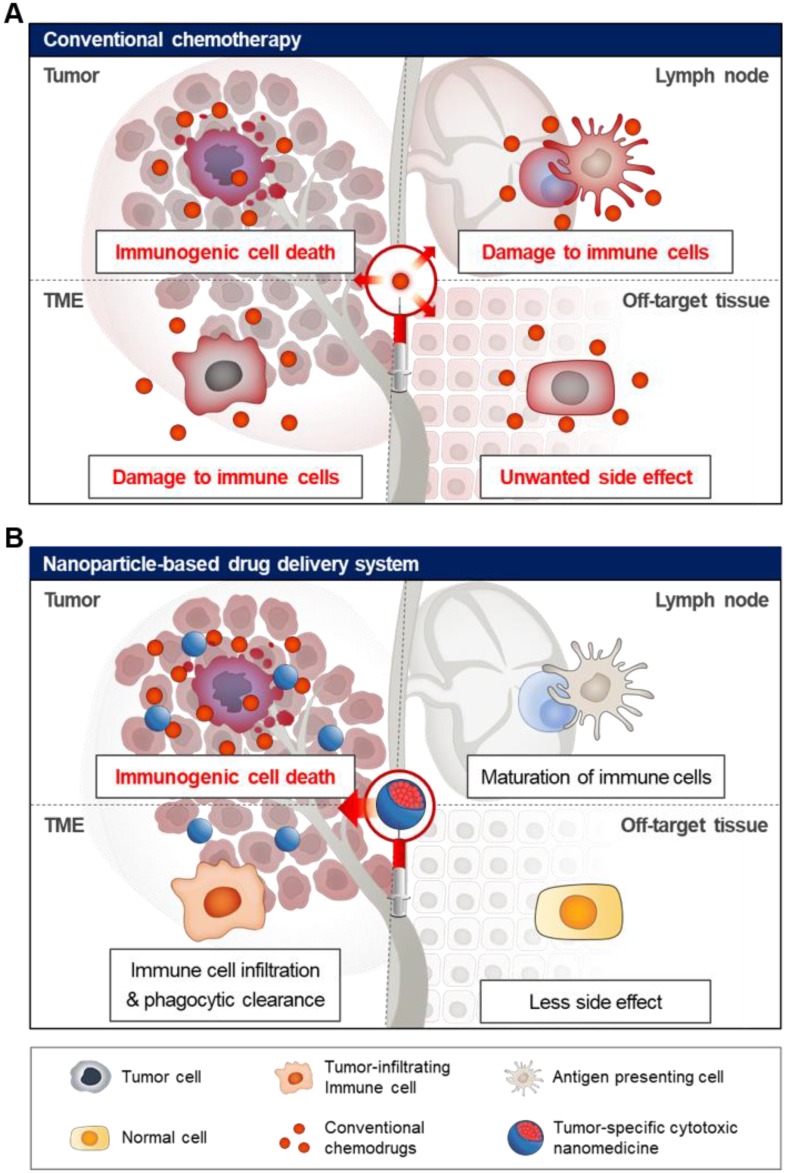
Schematic illustration of off-target toxicity in conventional chemotherapy. (A) Chemotherapy generally acts by killing fast-growing cells including malignant tumor cells and normal cells, leading to high off-target toxicity. (B) Nanoparticle-based drug delivery system can be used for tumor-specific delivery of chemotherapeutic drugs using the EPR effect and active targeting strategy, resulting in a significant increase in drug levels in the tumor tissue. Finally, cytotoxic chemotherapy induces tumor-specific immunogenic cell death in the tumor tissue, activating immune response with fewer side effects.

**Figure 3:**
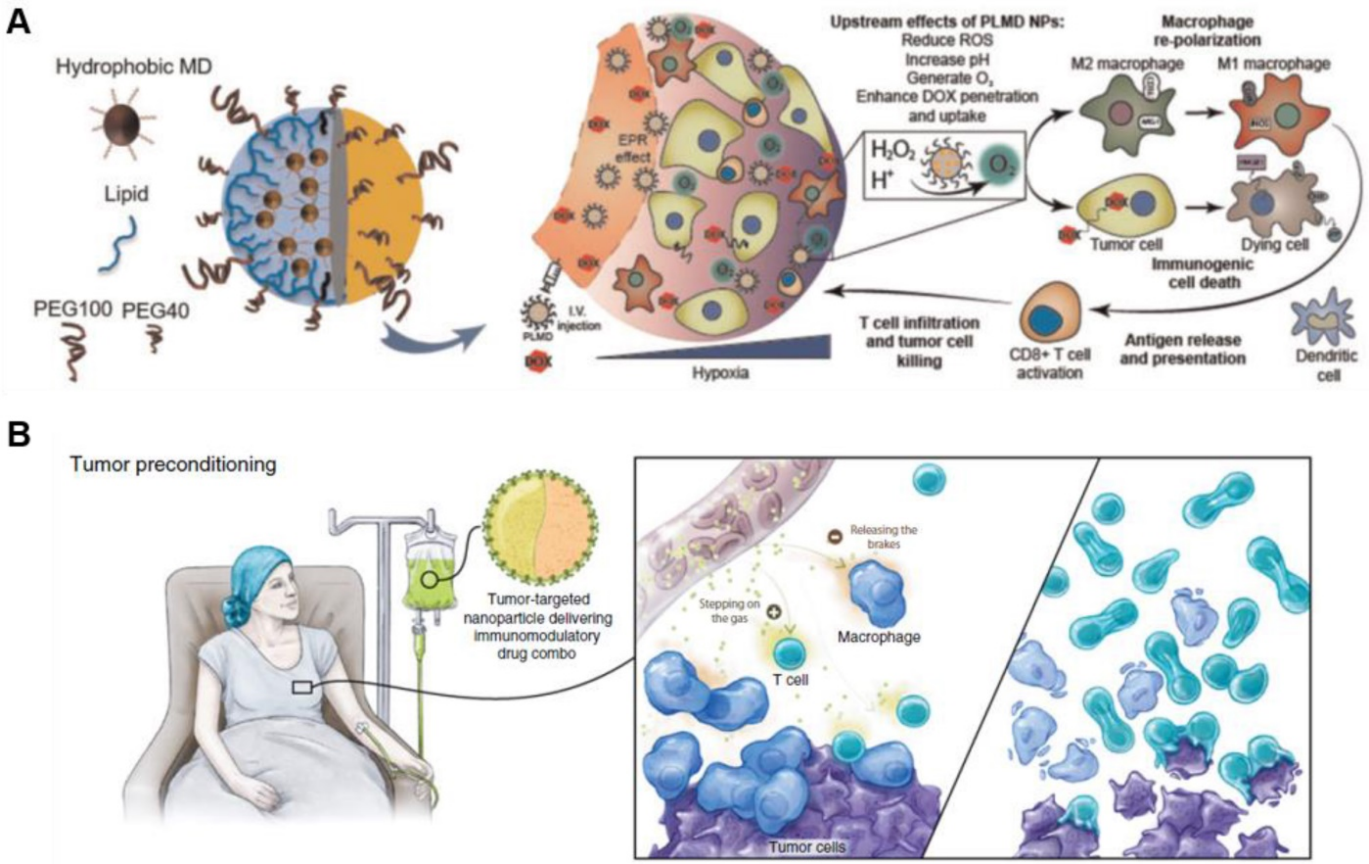
Modulation of functional nanoparticles with anti-cancer agents enhances therapeutic efficacy and boosts antitumor immunity. (A) Treatment using PLMD combined with doxorubicin leads to accumulation in the tumor tissue via EPR effect, reducing hypoxia and acidosis. More importantly, it enhances doxorubicin-induced immunogenic cell death-promoting macrophage phenotype polarization from M1 to M2 type. Adapted with permission from 43, Copyright 2019 Oxford University Press. (B) The schematic illustration shows that targeted nanoparticles potentially improve immunotherapy by remodeling TME and stimulating key antitumor effector cells. Adapted with permission from 44, Copyright 2018 AACR publications.

Conventional immunotherapeutic agents including immune checkpoint inhibitors fail to completely eliminate most types of tumors in the body, and show limited therapeutic efficacy 25, 26. Clinical studies have shown that only a fraction (10-38%) of cancer patients respond to current treatment using immune checkpoint inhibitors 27-31. Additional synergistic mechanisms are needed to improve the therapeutic efficacy of current immunotherapy of cancer patients co-administered with pre-existing immune checkpoint inhibitors or ICD-inducing cytotoxic drugs 27. Cytotoxic drugs such as doxorubicin, 5-fluorouracil, gemcitabine, paclitaxel, mitoxantrone and oxaliplatin elicit an immune response by activating apoptotic pathways and triggering ICD 32, 33. ICD in tumors induced with cytotoxic drugs triggers an antitumor immune response, leading to activation of the immune system against tumors 34, 35. The ICD in the tumor is characterized by damage-associated molecular patterns (DAMPs), including overexpressed high mobility group protein B1 (HMGB1), cell surface-exposed calreticulin (CRT) and adenosine triphosphate (ATP) 36, 37. In particular, surface‐exposed CRT and secreted ATP regulate immunogenicity in chemotherapy-induced apoptosis. Also, crucial DAMPs such as extracellular HMGB1 contribute to enhanced ICD by binding to toll-like receptor 4 (TLR4) on dendritic cells, potentiating cancer immunotherapy. In general, anticancer chemotherapy has a lethal effect on malignant cancer cells as well as other normal cells, leading to high off-target toxicity in normal and immune cells. Therefore, simple combination therapies comprising pre-existing cytotoxic drugs lead to severe clinical challenges such as unwanted systemic toxicity and immune suppression. In particular, repurposing NP-based drug delivery systems of ICD-inducing anticancer drugs facilitates cancer immunotherapy for maximal therapeutic efficacy with low toxicity. NP-based delivery of cytotoxic drugs showed enhanced drug efficacy and diminished unwanted off-target toxicity, due to their tumor targeting ability in many preclinical and clinical tests (**Figure 2**) 15, 38. The NP-based tumor-targeting delivery systems increased the retention time of cytotoxic agents at the tumor site and the rapid and enhanced cellular uptake of NPs boosted the tumor-specific immune response. It is well known that macromolecules including NPs and nanocomplexes enter the tumor interstitial space and accumulate in the tumor tissue, contributing to the EPR effect 39-41. Tumor-specific delivery of cytotoxic agents alter the immunosuppressive conditions in tumor tissues by facilitating tumor immunogenicity of antigens in many cancers and enhancing ICD to potentiate cancer immunotherapy 42. For example, polymer-lipid doxorubicin-encapsulated manganese dioxide NPs were successfully delivered to the tumor tissue due to the EPR effect, reversing immunosuppressive conditions in an orthotopic mouse model of breast tumor (**Figure 3A**) 43. In this study, attenuation of hypoxia and acidosis via NP treatment led to remodeling of the TME and boosted T-cell activity, inhibiting tumor progression. This type of tumor-specific delivery and immunogenic cytotoxic effect of NP-based delivery system may facilitate cancer immunotherapies or other T cell therapies used to treat tumors (**Figure 3B**) 44. Given the merits of tumor-specific EPR effect of NPs, ICD-inducing delivery platform can be explored to maximize the clinical outcomes of various immunotherapies. In addition to killing cancer cells, NP-based drug delivery systems for ICD-inducing cytotoxic drug can modulate immune reaction in TMEs, resulting in anti-tumor immune response 45. Several new developments have been reported in NP-based drug delivery systems to carry cytotoxic drugs to targeted cancer cells 46-49. This section focuses on recent advances in NP-based delivery systems of cytotoxic drugs leading to enhanced ICD in TME.

Recent studies show that novel NP-based delivery systems can be combined with immune modulators to enhance ICD response and antitumor immunity 50. For example, Zhang's group recently showed that nano-sized prodrugs consisting of doxorubicin, matrix metalloproteinase (MMP)-cleavable peptide and hyaluronic acid (HA) initiated antitumor immune response via upregulation of interferon-γ (IFN-γ) and programmed cell death protein-ligand 1 (PD-L1) 51. The prodrug-based NPs with a uniform size successfully induced the release of ICD-associated molecules, including HMGB1, IFN, and tumor necrosis factor (TNF) α in TME resulting in enhanced immune response by activated T cells. When combined with anti-PD-1 antibody (a-PD1), prodrug-based NPs elicited strong antitumor immune response and antitumor immunity, mediated by robust tumor-infiltrating lymphocytes (TILs). Thus, NP-based delivery systems carrying cytotoxic drugs can be used to facilitate ICD via mature antigen-presenting cells (APCs) in TMEs, However, the TME shows distinct mechanisms of immunosuppression, and infiltration by immunosuppressive cells, such as myeloid-derived suppressive cells, tumor-associated macrophages, and regulatory T cells 52. Repurposing the NP-based drug delivery systems of cytotoxic agents targeting the immune system may overcome the lower efficacy and potential toxicity of current cancer immunotherapeutics. To boost the ICD response and increase the antitumor immunity, immunosuppressive pathway inhibitors may also be used together with chemotherapeutic drugs. Nel et al. recently utilized mesoporous silica NPs (MSNPs) with oxaliplatin (OX) and immunosuppressive indoleamine 2,3-dioxygenase (IDO) pathway inhibitor (indoximod; IND) to treat orthotopic pancreatic ductal adenocarcinoma 53. In the study, the MSNPs induced ICD successfully and recruited several cytotoxic T lymphocytes in TME, resulting in induction of antitumor immunity. The subsequent release of DAMPs including HMGB-1 and ATP from tumor cells by the action of NPs provided immunogenic stimuli to the antigen presenting DC. Also, CRT expression on the dying cell surfaces in TME promoted an “eat-me” signal for the cellular uptake of DCs, which resulted in significant enhancement of the ICD effect by the IND. This study indicated that the combination therapy using NP-based delivery system containing both OX and IND resulted in a synergetic immunotherapy response by enabling induction of ICD along with a reversal of immune-suppressive effects. In addition, MSNPs improved the pharmacokinetics (PK) and tissue distribution of both OX and IND, which may increase the antitumor immunity synergistically.

Various NP-based drug delivery systems containing chemotherapeutic agents have successfully modulated the immune response against tumors via EPR effect or active targeting mechanism, compared with free chemotherapeutic agents. These NPs containing polymers, inorganic, and bio-derived nanomaterials effectively deliver immune-modulating agents into tumors 54. In particular, exosomes are the representative endogenous NPs that play a great role in the delivery of anticancer drugs combined with immune modulators because they contain various immune-modulating cytokines derived from cells 54, 55. For example, exosomes isolated from M1- macrophages directly enhanced the antitumor efficacy of chemotherapeutics in TME 56. In this case, the antitumor efficacy of paclitaxel (PTX) was significantly improved in tumor-bearing mice following simultaneous delivery of ICD-inducing PTX and pro-inflammatory cytokines to the targeted tumor site by the exosomes. The TME-activated binary cooperative NP with acidity-induced cleavage of poly(ethylene glycol) shell and glutathione-mediated linker is another new NP-based delivery system used to modulate immunosuppressive TME. The enhanced accumulation of self-assembled cytotoxic prodrugs in TME triggered ICD of targeted cancer cells 57. In the study, the tumor-specific action of dual‐activatable prodrug NPs elicited antitumor immunity resulting in enhanced immunotherapy, and intratumoral accumulation of cytotoxic T lymphocytes. The TME-specific nano-prodrugs exhibit great potential for clinical application by effectively delivering TME-activated prodrug to targeted tumor cells, resulted in enhanced immunogenicity of tumor cells without affecting normal immune cells.

Lastly, several studies showed that encapsulation of a cytotoxic agent in NPs eliminated undesirable immune side effects of peptides and liposomes. For example, drug delivery using encapsulated cytotoxic agent (doxorubicin) completely eliminated the lethal immunotoxicity of peptides and liposomes in ICR (Institute of cancer research) mice 58. Lu et al reported that cyclic RGD peptide-based modification of liposomes induced acute systemic anaphylaxis, IgG immune complex-triggered complement activation, and cytokine release. Minimizing the ratio of cyclic RGD peptide, or decreasing the injection doses failed to resolve the acute systemic toxicity. However, encapsulated NPs of doxorubicin eliminated the systemic immune response by inhibiting immunotoxicity and antibody overproduction. Therefore, unwanted immunotoxicity of NPs can be controlled by encapsulation of cytotoxic drugs. Recent advances in NP-based delivery systems, therefore, increase therapeutic efficacy by triggering ICD in TME, and decreasing systemic toxicity by TME-specific delivery 15.

Recent advances demonstrated a variety of applications in cancer immunotherapy via targeted delivery of ICD-inducing agents with adjuvants. Furthermore, by delivering various immunotherapeutic agents with synergistic effect, they contribute to an immunogenic TME, resulting in antitumor immunity. However, the poor drug delivery efficiency and systemic toxicity of NP-based systems in cancer immunotherapy are still persistent challenges limiting their clinical application. Therefore, the tumor-specific design of NPs, which can improve the delivery of cytotoxic agents is crucial for successful cancer immunotherapy and development of immunostimulating agents for treatment of cancer patients.

## 3. Advanced NP-based drug delivery systems of cytokines and cytokine-like immune modulators

Cytokines are a broad category of small biomolecules that are important in cell signaling. In cancer therapy, among cytokines, IFNs, interleukins, and chemokines have been widely used as immunomodulating agents. Specifically, IFN-α, a representative FDA-approved cytokine has been used in the clinic to treat leukemia since 1986, and subsequently, recombinant interleukin-2 (IL-2) has been developed for cancer immunotherapy since 1992 59-61. However, these cytokines have short half-lives and limited stability, which can be overcome by using NP-based delivery systems 62, 63. NP-based cytokine delivery can overcome limitations of conventional cytokine-based therapy such as short half-life, autoimmune attack and inflammatory immune reaction 64-67. In addition, several NPs have shown potential to modulate and fine-tune inflammatory responses to facilitate their use as immune-modulating agents in anti-cancer treatment. Various studies have shown that cytokines are easily encapsulated or chemically conjugated to different NPs, preventing degradation of cytokines by enzymes *in vivo* . Conjugation of cytokines on the surface of NPs enables cytokine delivery to the target receptors on the cell surface. Moreover, NPs selectively deliver cytokines to target tissues via controlled release mechanisms 66, 68, 69. For example, conjugation of IL-2 as T cell growth factor on the surface of hydroxyethyl starch nanocapsules (HES-D-IL-2) via copper-free click chemistry increased the binding affinity of IL-2 receptor compared with free IL-2, and enhanced the targeting efficiency of T cell populations 68. In addition, T cell immune response is closely dependent on the number of conjugated IL-2 molecules on the nanocapsule surface. HES-D-IL-2 nanocapsules are significantly absorbed by activated CD4^+^ CD25^+^ T cells compared with naïve CD4^+^ CD25^-^ T cells, resulting in enhancing activated T cell proliferation. This study indicates that the immune response of activated T cells can be modulated by controlling a number of ligands on the nanocapsule surface. Similarly, conjugation of tumor necrosis factor related apoptosis-inducing ligand (TRAIL) with the lipid nanocarrier membrane enhanced the potency of cytokines in cancer therapy 69. The expression of TRAIL on the surface of the lipid nanocarriers effectively enhanced the pro-apoptotic activity of extrinsic TRAIL pathway and increased the activity of apoptosis-inducing caspases. Immunotherapeutic approaches using NP-based delivery system usually improved the targeting efficiency and PK/pharmacodynamics (PD) of encapsulated or conjugated cytokines *in vivo* 67, 70-73. In order to maximize the therapeutic efficacy of cytokines, some NPs with cytokine-like ability have been developed in cancer immunotherapy. In this regard, recently developed NPs have been surface-modified using two or more surface moieties to investigate their role as cytokines. In particular, the development of a NP with two distinct faces (Janus-faced) and functions, represents a new direction for cancer immunotherapy 74. The control of immune activity using multifaceted NPs suggests an immunomodulatory role. Therefore, in this section, we described recent advances in cytokine-like immune-modulating NPs as novel NP-based delivery systems in cancer immunotherapy.

Multifaceted NP platforms improve the selective delivery and targeting ability of many cytokine-like immune-modulating agents to target cancer and immune cells with efficacy. A variety of studies report the development of NPs by engineering cytokines via chemical conjugation or physical loading of various immune modulators such as antibodies, immune-related proteins and genes 67, 75, 76. Multifaceted NPs containing two or more immune-modulating agents, which selectively target tumor tissues and inhibit tumor growth, have the potential to enhance antitumor immunity. Furthermore, the NPs are involved in immunotherapy as agonists or antagonists that specifically modulate the complex cancer immunity. Recently, multifaceted NPs have facilitated simultaneous targeting of lymphocytes and cancer cells. The Schneck's group developed NPs, stimulating T cells to kill tumor cells, by coating a single NP with tumor cell-binding antibody (a-human CD19) and loaded antigen-specific T cell-binding peptide (MHC-binding peptide) 75. The multifaceted NPs were selectively targeted to tumor cells to stimulate antigen-specific T cells, which resulted in tumor regression. Moreover, multifaceted NPs readily control the complex binding affinities to different targeted cells by altering the ratios of various tumor antigens on the NP surface and boost the lethal effect of T cells against tumors. Kim et al. reported tumor eradication using multifaceted NPs by simultaneous targeting cancer cell-specific receptors and signaling phagocytosis of macrophages 77. The multifaceted NP specifically targeted cancer cells overexpressing human epidermal growth factor receptor-2 and strongly stimulated professional APCs by calreticulin, which is a protein inducing phagocytosis. Consequently, phagocytosis by APCs was triggered by multifaced NPs, which are selectively bound to cancer cells, resulting in innate and adaptive immunity.

**Figure 4:**
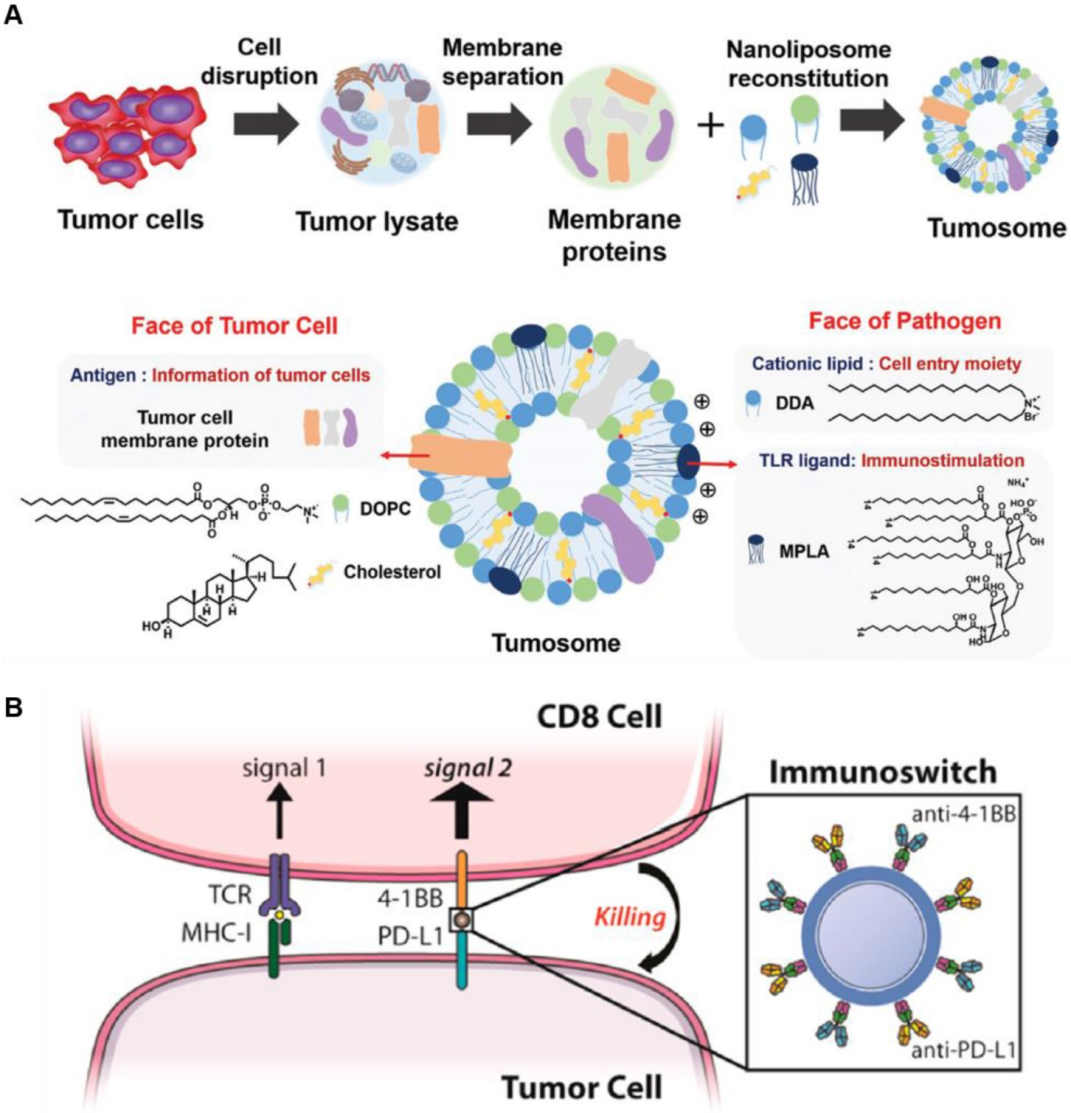
Schematic illustration of multifaceted immune-modulating nanoparticles and immunoswitch nanoparticles. (A) Multifaceted nanocarriers derived from tumor cells are composed of tumor cell membrane proteins including tumor antigens and pathogen ligand, which act as immunostimulatory adjuvants. Adapted with permission from 79 copyright 2017 Wiley-VCH. (B) Multifaced nanoparticles act as immunoswitches coated with two types antibodies (anti-4-1BB and anti-PD-L1). The anti-4-1BB efficiently induces a T cell response and anti-PD-L1 significantly blocks the immunosuppressive pathway of tumor cells. Adapted with permission from 76 copyright 2017 American Chemical Society.

As an interesting multifaceted NP platform, cell-derived nanovesicles represent intracellular immune-modulating NPs that potentially enhance antitumor immunity 78, 79. For example, multifaceted nanovesicles derived from macrophages or tumor cells were synthesized to boost endogenous immune response. M1 macrophage-derived nanovesicles effectively encapsulated M1 macrophage markers and mRNAs of pro-inflammatory factors, and replicated the function of M1 macrophages during immunotherapy 78. The M1 macrophage-derived nanovesicles efficiently polarized M2 tumor-associated macrophages to antitumor M1 type macrophage and not only enhanced the secretion of antitumor cytokines but also significant suppressed the tumor growth. Alternatively, tumor cells derived from multifaceted nanovesicles have the potential to deliver tumor antigens and adjuvants to LNs resulting in an enhanced antitumor immune response 79. The tumor cell-derived nanovesicle was composed of various proteins in the tumor cell membrane acting as tumor antigens, and a pathogen ligand, which facilitated antigen recognition by APCs (**Figure 4A**). In this study, multifaceted nanocarriers showed that incorporating the NPs with various immunomodulating agents, such as cancer cell-specific biomarkers, triggers an adaptive immune response.

The immunosuppressive TME hampers immune checkpoint inhibitor therapy and promotes metastasis of cancer cells 76, 78, 80. The multifaceted NP platform can be used to overcome these limitations by modulating TME or targeting metastatic cells in the circulation. Schneck's group developed dual-targeting NPs, which simultaneously stimulate immune cells and block immune checkpoints on cancer cells. The multifaceted NPs were coated with two types of antibodies (anti-4-1BB and anti-PD-L1). The anti-4-1BB induces tumor-targeted CD8^+^ T cells to increase the secretion of cytokines including IFN-γ. The anti-PD-L1 significantly blocks the immunosuppressive pathway of cancer cells to prevent immune evasion from cytotoxic T cells (**Figure 4B**) 76. Combination therapy with both antibodies using the NP platform significantly enhanced the therapeutic efficacy compared with conventional treatment using soluble anti-PD-L1 and anti-4-1BB. This study showed that multifaceted NPs reduce immunosuppression in the TME and stimulate the activity of T cells for effective cancer immunotherapy. Recently, King et al. developed multifaceted NPs coated with TRAIL and E-selectin adhesion molecules to target metastatic cancer cells 81. E-selectin on NP surface specifically binds to leukocyte membrane, and therefore, TRAIL is circulated in the blood by attached to white blood cells and can evade renal clearance. By tethering NPs to leukocytes, this approach mimics the cytotoxic activity of tumor-targeted T cells, and effectively eradicates metastatic cancer cells from the blood. Taken together, the multifaceted NP platform, which has a great potential for specific targeting, reduces off-target effects while enhancing the efficacy of antitumor activity. Furthermore, multifaceted NPs can deliver various immune modulators, which act as cytokines in cancer immunotherapy.

**Figure 5:**
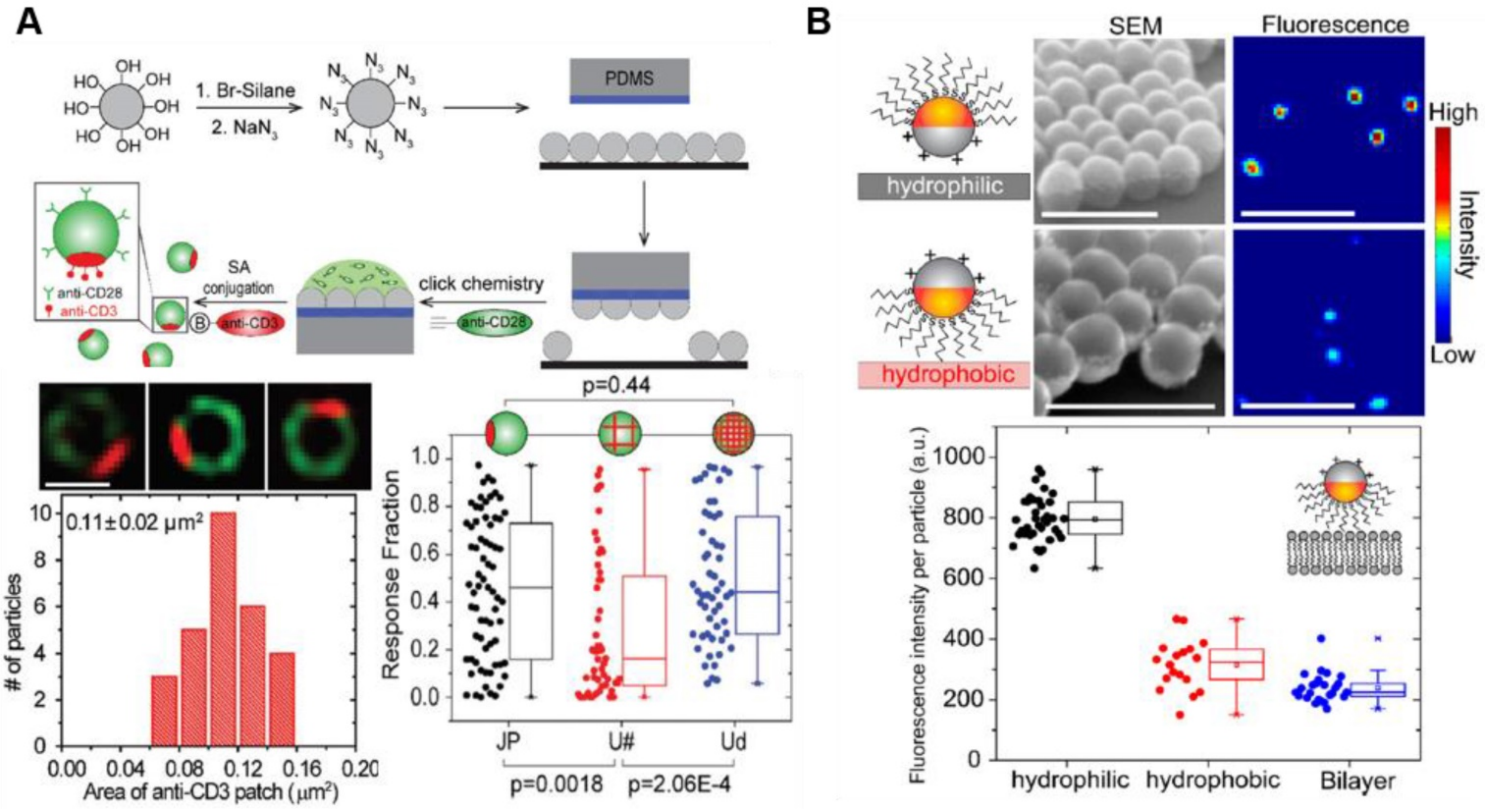
Utilization of Janus nanoparticles for cancer immunotherapy. (A) Janus nanoparticles were functionalized with alkyne-tagged anti-CD28 antibodies via azide groups via click chemistry. Super-resolution fluorescence images of the Janus nanoparticle show spatial segregation of anti-CD3 (red) and anti-CD28 (green) clusters in the nanoparticle. The spatial arrangement of ligands in the Janus nanoparticles facilitates simultaneous binding events including T cell activation. Adapted with permission from 83, Copyright 2017 The Royal Society of Chemistry. (B) SEM and fluorescence images of the Janus nanoparticles show the orientation on hydrophilic and hydrophobic substrates. The 100 nm Janus nanoparticles successfully induced defects in zwitterionic lipid bilayers. Adapted with permission from 84, Copyright 2018 ACS publications.

Recent studies have shown that a single NP with different surface characteristics exhibits a range of cytokine-like activities in cancer therapy 82. Among multifaceted NP-based delivery systems, Janus NPs carrying surfaces with two or more distinct biochemical properties have been used to modulate NP-cell interactions similar to natural cytokines. Recent advances in cancer immunotherapy suggest that Janus NPs have a great potential in immune cell response. For example, Janus NPs with an increased surface density of ligands activated T cells significantly compared with uniformly coated NPs carrying the same number of ligands 83. The spatial arrangement of ligands in the Janus NPs may affect simultaneous binding events including T cell activation for cancer immunotherapy, providing a new strategy to increase co-stimulatory immune response and T cell stimulation (**Figure 5A**) 83. Until now, this approach was not used to design immune-modulating drugs due to the technical challenges associated with the fabrication of biomolecule clusters on NP surfaces. Recent advances in nanotechnology have enabled the design and synthesis of specialized functional NPs facilitating interaction with various immune cells. For example, Yu et al. showed that amphiphilic Janus NPs with different charges or hydrophobicity disrupted lipid bilayers more effectively than other uniformly coated NPs (**Figure 5B**) 84. The Janus NPs measuring 100 nm in size induced defects in zwitterionic lipid bilayers at picomolar concentrations. Owing to their extraordinary heterostructure and surface modulation, they have been used in drug delivery associated with cancer immunotherapy 85, 86. Designing NPs with distinct structural features and unique functionalities may provide a new approach in immunotherapy.

Recent studies showed that NPs effectively deliver cytokines and cytokine-like immune modulators to target cells, resulting in improving therapeutic efficacy as well as prolonged half-life and stability. However, the rational design of NPs requires reducing off-target effects and cytokine resistance for clinical application.

## 4. Advanced NP-based drug delivery systems of adjuvants

In cancer immunotherapy, adjuvants play a significant role in activating the APCs and generating a strong immune response 87, 88. Thus, adjuvants have been widely used to formulate vaccines to enhance the immunogenicity of antigens and vaccine efficacy. However, adjuvants increase the risk of unwanted severe toxicity. Indeed, very few adjuvants have been approved for human use because of severe toxicity. Repurposing the NP-based delivery systems of adjuvants has been shown to significantly contribute to enhanced immunogenicity and diminished toxicity. In addition, NP-based adjuvant delivery with antigen can be used to channel the immune response either toward T helper cell type 1 (Th1) or T helper cell type 2 (Th2) 89.

Co-delivery of adjuvant and antigen is an effective therapeutic strategy in adjuvant-based immunotherapy 90-92. Particle-based vaccines can be used to co-deliver adjuvant and antigen to the same APCs for selective uptake the antigen, using a relatively low amount of adjuvant for efficient immune response and reduce the risk of unwanted toxicity 93. Furthermore, co-delivery of adjuvant and antigen using particle-based vaccine has been shown to promote antigen cross-presentation and cytotoxic CD8^+^ T cell activation 87. The commonly used adjuvants in NPs for cancer immunotherapy include aluminum salts (alum), lipopolysaccharide (LPS), CpG oligodeoxynucleotides (CpG ODNs), layered double hydroxide (LDH), and polyinosinic:polycytidylic acid (poly I:C) 94, 95.

**Figure 6:**
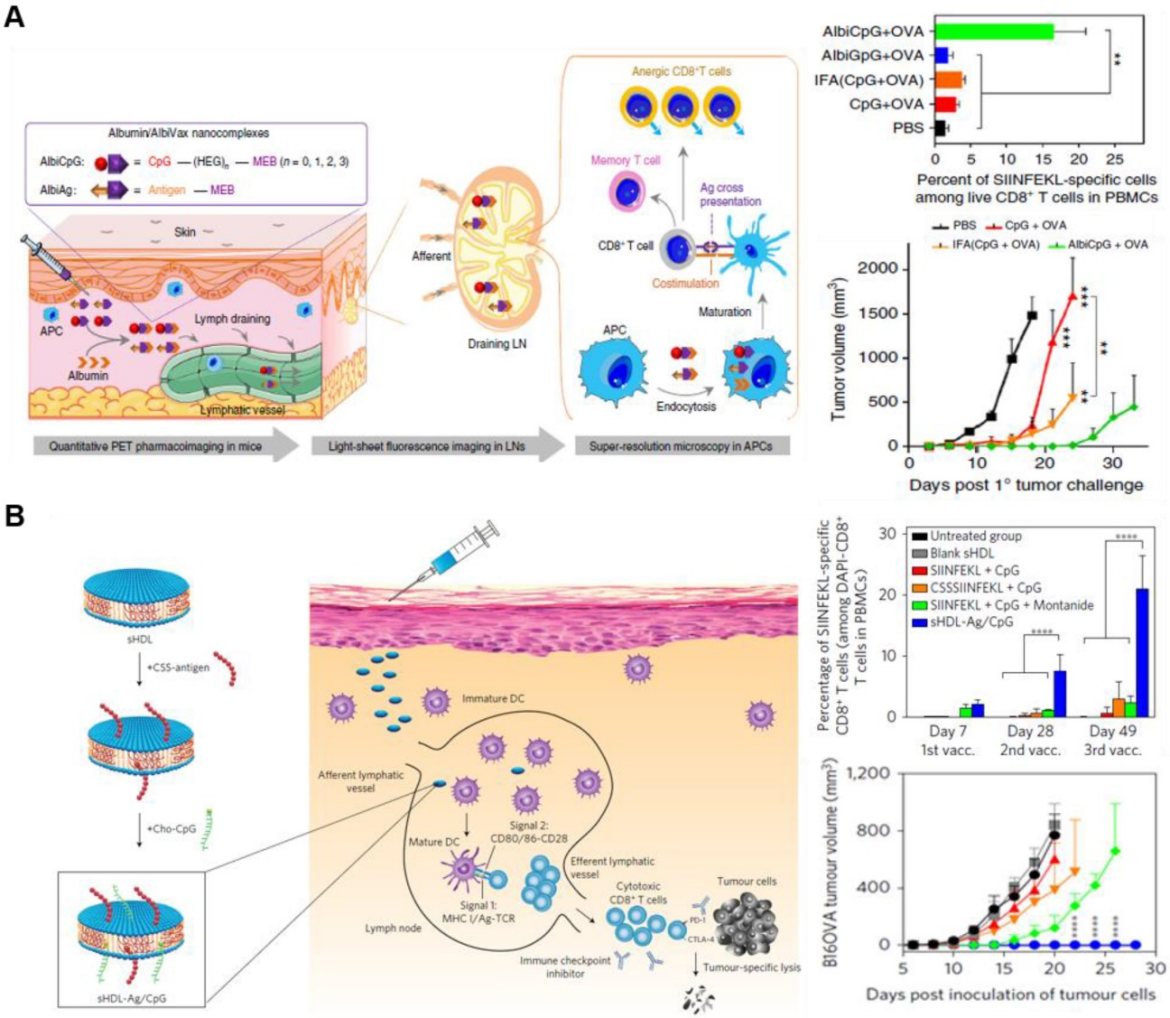
Schematic illustration of nanoparticle-based delivery strategies of adjuvant and antigen for efficient immune stimulation. (A) Albumin/AlbiVax nanocomplexes composed of AlbiCpG and AlbiAg bind to endogenous albumin *in vivo* . Albumin/AlbiVax nanocomplexes efficiently activated APCs inducing antitumor immunity by stimulating CD8^+^ T cells. Adapted with permission from 97 Copyright 2017, Springer Nature. (B) Synthetic sHDL nanodisk-based adjuvant and antigen co-delivery system. The sHDL nanodisks are composed of adjuvant CpG and tumor-specific neoantigens, which can be delivered to LNs. The sHDL nanodisks were absorbed by DCs followed by antigen cross-presentation. Finally, cytotoxic T cells were significantly activated to kill tumor cells. Adapted with permission from 98 Copyright 2017, Springer Nature.

The adjuvant and antigen can be co-delivered in separate NPs or in a single NP. For example, Lim's group developed interesting synthetic vaccine NPs (SVNPs) that target LNs and enhance antitumor immunity 96. SVNPs are composed of two types of NPs: one carrying the adjuvant, a toll-like receptor 3 (TLR3) agonist (poly I:C), and the other carrying the model tumor antigen, ovalbumin (OVA) prepared by electrostatic adsorption. Poly I:C is a double-stranded RNA and interacts with TLR3, which are expressed on leukocyte membranes. SVNPs showed higher cellular uptake into APCs, enhancing the secretion of type Ⅰ IFN-α and -β and pro-inflammatory cytokines compared with soluble poly I:C or OVA. Furthermore, Chen's group recently developed a noncomplex platform for efficient co-delivery of adjuvant and antigen using endogenous albumin. The albumin-binding vaccine (AlbiVax), consisting of albumin-binding adjuvant (AlbiCpG) and antigen (AlbiAg), is self-assembled with endogenous albumin *in vivo* (**Figure 6A**) 97. The AlbiVax-based nanocomplexes were efficiently delivered to LNs and induced antigen-specific T cell responses in mice. Immunotherapy using albumin/AlbiVax nanocomplexes significantly enhanced the therapeutic effect. The adjuvant and antigen can also be co-delivered by loading them in the same nanocarrier. Moon *et al*. have shown that using synthetic high-density lipoprotein (sHDL) as a nanocarrier combined with adjuvant and neoantigen significantly improved the adjuvant delivery to LNs and antigen presentation on APCs (**Figure 6B**) 98. Moreover, sHDL delivered multi-epitope antigens that induced broad T cell responses, resulting in enhanced tumor immunotherapy. Similarly, the combination of sHDL with adjuvants leads to a strong immune response resulting in effective tumor regression 99. Further studies are needed to evaluate the co-delivery via different loading methods to determine the most effective targeting technique of immune cells and LNs. Studies are currently ongoing to investigate the ways to increase the effectiveness of immunotherapy by NPs depending on the formulation of adjuvant and antigen 100-102. Groettrup *et al*. demonstrated that the immunogenicity of the encapsulated antigen and adjuvant in the same carrier yielded a higher immune response than those encapsulated in separate carriers 100, 103. A biodegradable poly (D, L-lactide-*co*-glycolide) carrier was designed to co-deliver CpG ODNs or poly I:C with OVA antigen. Although both co-delivery methods using same or separate carrier enhanced T cell immunity, encapsulation within the same carrier strengthened the cytotoxic T lymphocyte response and further enhanced the cytokine secretion. Conversely, another recent study showed that separately loaded adjuvant and antigen resulted in greater or similar effects of immune therapy compared with similar carriers 102. The CpG adjuvant was encapsulated into virus-like particles (VLPs), and p33 antigen was chemically conjugated on the same or separate VLPs. Delivering CpG and p33 in separate VLPs yielded greater CD8^+^ T cell responses compared with those delivered by the same VLPs. Collectively, NP-based drug delivery systems facilitates co-delivery of adjuvant and antigen either in a single or separate particle although the potency of immune response induced is still disputed. In addition, the therapeutic efficacy of the vaccine varied depending on the loading method of adjuvant and antigen, which should be considered in the design of a carrier for co-delivery.

Recently, the effect of physicochemical properties, especially the size of adjuvant-containing carriers on immune activation has attracted great attention in vaccine development. In addition, adjuvant density on the carrier surface and particle shape have been investigated 22, 92, 104-106. Nanotechnology can be used to alter the shape of carriers and the density of adjuvants on the carrier. Physicochemical properties of carriers with adjuvants affect the targeting to dendritic cells (DCs) and modulation of DCs 107, 108. During shape modification, alum-based adjuvants are commonly used in vaccines to stimulate the innate immune response 109-111. In order to improve the adjuvant effect of alum, the immunological effect according to physicochemical properties was studied by Xia's group using synthetic γ-phase aluminum oxyhydroxide NPs (γ-ALOOH) in the form of nanoscale plates, polyhedra and various rod sizes 95. In this report, ALOOH nanoplates and nanopolyhedra showed a lower level of cytokine expression than commercial alum, but ALOOH nanorods boosting the immune responses compared with the commercial alum. ALOOH nanorods enhanced NOD-, LRR- and pyrin domain-containing protein 3 (NLRP3) inflammasome activation and enhanced the release of pro-inflammatory cytokine IL-1β. The nanorods of smaller hydrodynamic size showed few *in vivo* immune responses among the various nanorods investigated. Notably, in another study, Niikura *et al*. demonstrated that the size and shape of particle influenced the adjuvant and immune responses depending on the injection method 112. During intranasal administration, the shape of gold nanorods attached to adjuvant enhanced the immune response compared with spherical nanorods without inducing excessive inflammatory responses. However, the subcutaneous administration did not significantly affect the immune response according to the shape of gold NPs. The effect of carrier shape should be considered along with the effect of carrier size and route of administration. The effect of adjuvant density on particles has been investigated to optimize immunotherapy outcomes 92, 113. Seder's group designed and evaluated various particles to enhance vaccine efficacy using TLR-7/8 agonist as an adjuvant 92. The TLR agonist can be used to selectively stimulate leukocytes including APCs and DCs to enhance antigen-specific T cell immune response. Increasing the density of TLR-7/8 agonist on polymer carrier strongly stimulated CD4^+^ and CD8^+^ T cell immunity more than the lower density of adjuvant. Furthermore, TLR-7/8 agonists conjugated with polymer particles were markedly absorbed by DCs compared with the same total dose of adjuvant on the small molecule or polymer coil. Increasing the uptake of TLR-7/8 agonists conjugated with polymer particle by DCs enhanced the cytokine secretion in draining LNs. Interestingly, Roy *et al*. designed four types of pathogen-like particles (PLPs) based on the density of CpG adjuvant (maximum or low density) and the size of PLPs (microparticle or NP) 113. DCs treated with a maximum density of CpG effectively increased the pro-inflammatory IL12p70 cytokine secretion, and also enhanced the amount of IFN-γ produced in CD4^+^ T cells that were co-cultured with DCs treated with PLPs at a maximum CpG density. In addition, IL4 secretion of CD4^+^T cells tended to decrease inversely with increasing concentration of CpG. IFN-γ is associated with Th1 differentiation, and IL4 is linked to Th2 differentiation. In addition, particle size skews the immune response. Micro-sized PLPs potentially stimulate pro-inflammatory cytokine release from DCs, resulting in skewing to Th1. Conversely, nano-sized PLPs can simultaneously skew immune response to either Th1 or Th2 depending on the CpG concentration on the particles. Accordingly, CpG density and particle size can potentially program DCs, polarizing immune responses into either Th1 or Th2. Based on these studies, it is apparent that biophysical effects of adjuvant on the particles can regulate the immune system and dictate the type of immune response. Therefore, biophysical attributes such as size or shape of the carriers and ligand density in particle design should be considered during the development of an effective cancer vaccine.

Recently, various strategies using NP-based delivery system have been developed to deliver adjuvant and antigen for effective antigen cross-presentation and enhanced immune response. NP-based antigen/adjuvant delivery system improved the preclinical outcomes compared with conventional soluble adjuvant/antigen treatment by enhancing target specificity and delivery efficiency. Furthermore, appropriate modification of biophysical properties of NPs facilitated delivery of adjuvant/antigen via various routes of administration, which improved the immune response. However, the mechanisms underlying the changes in immune system based on the biophysical properties of antigen-presenting NPs are still unclear. Furthermore, the physicochemical properties of NPs need to be optimized to avoid unintended polarization of immune cells. Finally, a scale-up manufacture and personalized design of adjuvant/antigen using NPs can enhance their potential therapeutic efficacy for clinical application.

## 5. Perspective and Conclusion

**Figure 7:**
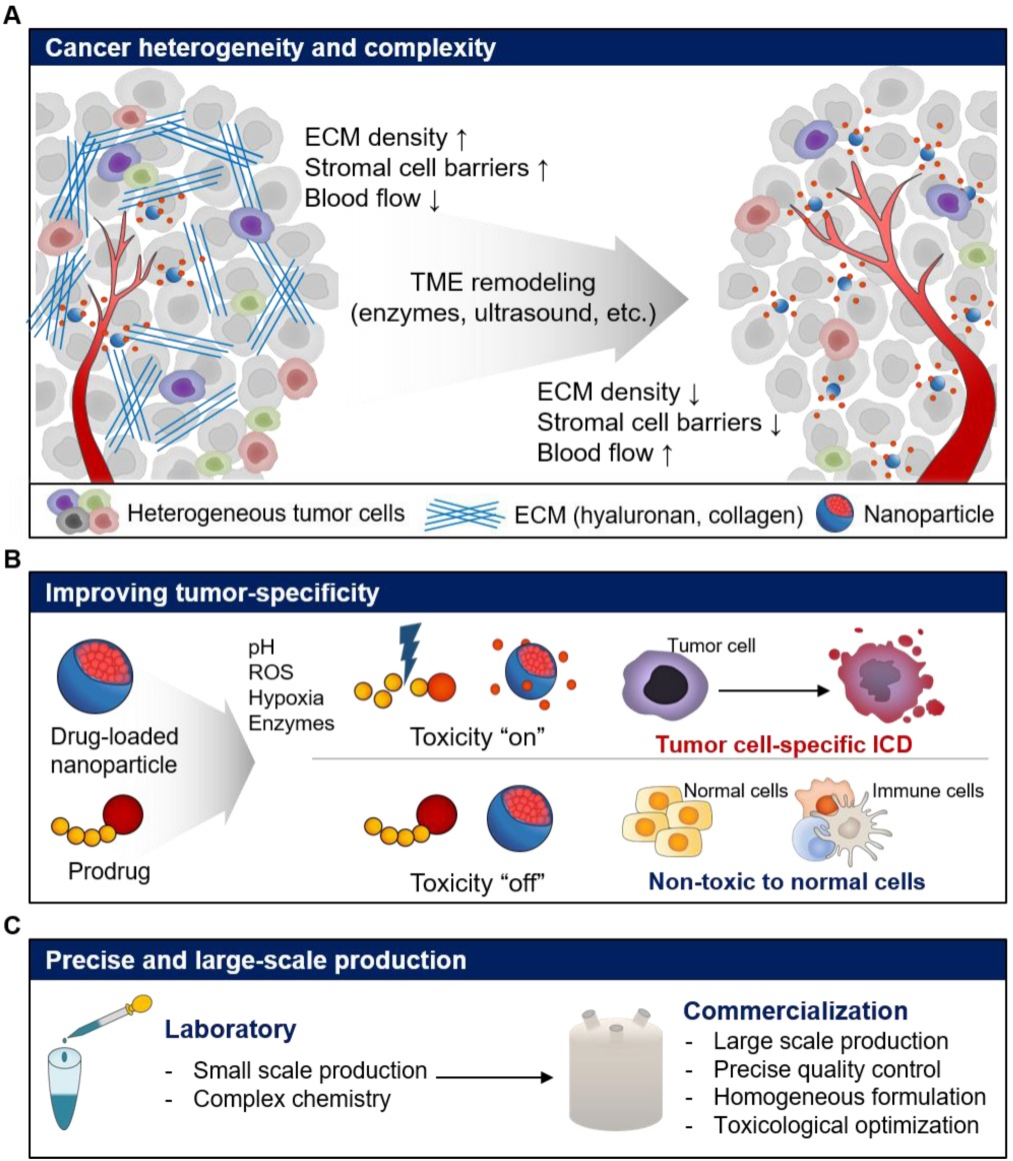
Expected challenges and perspectives for the repurposing of current nanoparticle-based drug delivery systems for cancer immunotherapy. (A) Repurposing strategies to overcome cancer heterogeneity and complexity for improved drug delivery efficiency. (B) Repurposing strategies improved tumor specificity of immunotherapeutics by activating immune responses with fewer side effects. (C) Repurposing strategies for precise and large-scale commercial production of immunotherapeutics.

In this review, we discussed the different issues for repurposing of current NP-based drug delivery systems for cancer immunotherapy. Recent advances in NP-based drug delivery systems have enhanced the delivery of immunotherapeutics such as antigens, adjuvants, immune checkpoint inhibitors, cytokines, and cytotoxic anticancer agents by repurposing the current NP-based drug delivery systems, resulting in improved anticancer immune response (**Table 1**). However, there are a number of limitations and challenges, which need to be overcome, to repurpose NP-based drug delivery systems for the delivery of immunotherapeutics to tumors (**Table 2**). Inherent properties of tumor, such as complexity and heterogeneity, can reduce the efficiency of NP-based drug delivery 114. The complex TME such as poor blood flow, stromal cell barriers, and dense ECM can interfere with tissue penetration of NPs, leading to reduced delivery efficiency. Furthermore, the targeting efficiency of NP-based drug delivery systems might vary between tumors due to the heterogeneous TME. Therefore, the delivery challenges need to be addressed to achieve the desired therapeutic efficacy of repurposed NP-based drug delivery systems (**Figure 7A**). Several groups reported that ECM remodeling strategy using enzymes such as hyaluronidase, collagenase, bromelain and matrix metalloproteinase, which degrade ECM in the tumor tissue, may enhance the delivery and tissue permeability of anticancer drugs and NPs 115-121. For example, PEGylated form of recombinant human hyaluronidase (PEGPH20), which removes hyaluronan from the ECM of solid tumors showed a significant increase in microvessels and reduced interstitial fluid pressure 122. In combination with anticancer drug, gemcitabine, PEGPH20 significantly inhibited the tumor progression and incidence of metastasis in a phase I clinical study 123. Treatment of patients with hyaluronan high (HA-high) metastatic pancreatic ductal adenocarcinoma with a combination of PEGPH20 and pembrolizumab is also in phase II clinical study. Cheng et al. found that recombinant PH20-embedded HPEG-PH20 NPs showed deep tissue penetration in 4T1 breast tumor 124. Notably, HPEG-PH20 NPs degraded HA matrix in tumor tissue, resulting in deep tissue delivery of DOX. Finally, HPEG-PH20 NPs prolonged the life span of tumor-bearing mice via successful inhibition of tumor growth. Therefore, versatile combinations and designs that can overcome these hurdles are needed to repurpose the current NP-based drug delivery systems for cancer immunotherapy.

To maximize the efficacy and minimize the toxicity in cancer immunotherapy, the target specificity and PK/PD of immunotherapeutics need to be carefully modulated, especially when cytotoxic drugs are used. Current NP-based drug delivery systems have shown potential for improved anticancer effect in recent decades. However, poor efficiency of drug encapsulation, innate toxicity and unintended release of cytotoxic drugs have reduced the therapeutic efficacy and resulted in adverse side effects 125, 126. To overcome these limitations and induce ICD using cytotoxic drugs, the TME including enzymes, pH, reactive oxygen species (ROS), and hypoxic conditions can be used to design tumor cell-specific cytotoxic drugs (**Figure 7B**) 127-130. For example, we designed cathepsin B-specific carrier-free prodrug NP (FRRG-DOX) using cathepsin B-cleavable peptide (Phe-Arg-Arg-Gly; FRRG) and doxorubicin for cancer targeting therapy 17. FRRG-DOX exhibited reduced toxicity in normal cells, but activated toxicity in tumor cells, which over-expressed cathepsin B. Furthermore, FRRG-DOX formed NPs without carriers, showing successful tumor-targeting and tumor-specific toxicity via EPR effect *in vivo.* We expect that repurposing tumor-specific prodrug designs can be used to induce tumor cell-specific ICD as well as chemotherapy resulting in improving antitumor immunity.

Beyond the tumor specificity of cytotoxic drugs, effective modulation of PK and the PD of the immune modulators including cytokines and immune checkpoint inhibitors is also needed to optimize the activation of immune responses in TME. Recombinant cytokines were approved for cancer immunotherapy; however, the short half-life of cytokines and cytokine release syndrome following high-dose bolus injection reduces the therapeutic efficacy 131. Also, cytokine treatment induces autoimmune reaction in normal tissue by promoting regulatory T cell survival and T cell death 132. Therefore, various approaches that improve target specificity and drug release are needed to repurpose the current NP-based drug delivery systems for optimal therapeutic efficacy and minimal toxicity. Finally, a relatively complex synthesis and quality control system is needed for the large-scale production of current NP-based drug delivery systems and their successful commercialization (**Figure 7C**). Thus, a precisely controlled and relatively simple chemistry suitable for large-scale production is needed to repurpose the current NP-based drug delivery systems. For example, a number of cancer vaccines with a synergistic effect have been developed. However, their clinical outcomes are unsatisfactory due to suboptimal conditions for large-scale manufacture, quality control, delivery efficiency of adjuvants and antigens to lymphoid organs, leading to weak immunostimulation and immune tolerance 61, 133, 134. To address these issues, Chen's group designed nanovaccines (AlbiVax) that form nanocomplexes (Albumin/AlbiVax) with endogenous albumin *in vivo* 97. In combination with anti-PD-1 or Abraxane, AlbiVax significantly inhibited tumor progression. Compared with conventional designs of nanovaccine, the endogenous albumin may have advantages including i) relatively simple and well-defined, chemistry-based, exogenous vaccine design to facilitate large-scale manufacture under stringent quality control, ii) antitumor immunity based on precise and effective delivery of nanovaccines to the target immune cells, resulting in enhanced delivery efficiency via albumin endocytosis, in which albumin receptors such as monocyte, DCs, and macrophages are highly expressed, and iii) increased PK of nanovaccines *in vivo* due to the albumin half-life *in vivo* 87. This study suggested a strategy to repurpose the current NP-based drug delivery systems for clinical translation, and relatively simple and well-defined large-scale production. A number of NPs related to cancer immunotherapy are currently in clinical trials after large-scale manufacturing process. Representative NPs in the translational stages are Lipovaxin-MM (Avg. 240 nm, IFN-γ with liposome) for malignant melanoma treatment (phase I, NCT01052142), Oncoquest-L (IL-2 with proteo-liposome) for lymphoma treatment (phase II, NCT02194751), and CYT004-MelQbG10 (A-type CpG with virus-like NP) for malignant melanoma treatment. Taken together, various NPs in immunotherapy have shown promise for further clinical application.

In this review, we summarized the recent advances for repurposing current NP-based drug delivery systems for cancer immunotherapy. Various designs and strategies of NP-based drug delivery systems were utilized to elicit antitumor immunity. Furthermore, the current limitations and challenges associated with NP-based drug delivery systems have been discussed, and future directions for nanomedicines to overcome these hurdles have been discussed. Using versatile strategies to overcome the current limitations, we expect that repurposing the current NP-based drug delivery systems provides an opportunity for successful cancer immunotherapy and further clinical applications.

## Figures and Tables

**Table 1:** Summarization of three categories of recent advances in NP-based delivery system for cancer immunotherapy

Category	Agent	Cell type	Average size	Composition	Method	Ref
ICD-inducing cytotoxic NPs	DTX	Glioblastoma cell (HF2303)	10-12 nm	ApoAI mimetic peptide, phospholipids and CpG	Self-assembly by nanodisc	37
	DOX	Breast cancer cell (EMT6)	170 nm	polymer-lipid based manganese dioxide NPs	Encapsulation	43
	DOX	Melanoma cell (B16F10)	250 nm	matrix metalloproteinase sensitive peptide (CPLGLAGG)	Self-assembly with hyaluronic acid	51
	Oxaliplatin	Pancreatic ductal adenocarcinoma (KPC)	80 nm	mesoporous silica NP with indoximod	Encapsulation	53
	PTX	Breast cancer cell (4T1)	170 nm	Nano-formulation Exosome with PTX	Loading into exosome	56
	DOX	Isolate lymphocyte	105 nm	c(RGDyK)-liposomes	Encapsulation	58
Cytokines and cytokine-like immune modulators NPs	IL-2	Melanoma cell (B16F10)	80 nm	PEGylated liposomes with anti-CD137	Surface-conjugated NP	46
	TRAIL	Colon adenocarcinoma (COLO 205)	100-140 nm	PEGylated liposomes with TRAIL	Surface-conjugated NP	69
	Peptide loaded MHC and anti-CD19	Lymphoblast cell (Raji)	50 nm	Anti-mouse IgG1 Microbeads with ^pep^MHC and anti-CD19	Surface-conjugated NP	75
	Anti-4-1BB and anti-PD-L1	Melanoma cell (B16 SIY)	80 nm	Antibiotin-coated iron-dextran with anti-4-1BB and anti-PD-L1	Surface-conjugated NP	76
	Anti-HER2 and CRT	Breast cancer cell (E0771)	45 nm	Carboxylated polystyrene NP with anti HER2 and CRT	Surface-conjugated NP	77
	M1 macrophages	Fibroblast cell (CT26)	190 nm	M1NVs and anti-PD-L1	Cell-derived nanovesicles	78
	TRAIL and E-selectin adhesion receptor	Colon adenocarcinoma (COLO 205)	118 nm	Multilamellar liposomes (PC and Chol)	Surface-conjugated NP	81
	Stimulatory ligands	Jurkat T cells	500 nm	anti-CD3 and anti-CD28 antibody	Conjugation with azide-functionalized silica NP	83
NPs for adjuvant delivery	layered double hydroxide (LDH)	Melanoma cell (B16F10)	140-150 nm	Tyrosinase-related protein 2 (Trp2) peptide	Adsorption and loading into LDH NP	94
	Alum	Human THP-1 myeloid cell	93-957 nm	γ-phase aluminum oxyhydroxide (γ-AlOOH)	Various size and shape of NP	95
	Poly I:C	Lymphoma cell (EG7-OVA)	20-70 nm	Poly(γ-glutamic acid), OVA	Self-assembly and electrostatic adsorption	96
	CpG	Lymphoma cell (EL4 and EG7-OVA)	10-20 nm	Albumin binding nanocomplexes, antigens (CSIINFEKL, Trp2, and Adpgk)	Chemical conjugation and self-assembly	97
	CpG	Melanoma cell (B16F10)	10 nm	Phospholipids, ApoA1-mimetic peptides, antigens (SIINFEKL, CSSSIINFEKL)	Self-assembly by nanodiscs	98

AlOOH: Aluminum hydroxide, ApoAI: Apolipoprotein-I, Chol: Cholesterol, CRT: Cell surface-exposed calreticulin, DOX: Doxorubicin, DTX: Docetaxel, ICD: Immunogenic cell death, IL-2: Interleukin 2, M1NVs: M1 macrophage-derived nanovesicles, NPs: Nanoparticles, OVA: Ovalbumin, PC: Phosphatidylcholine, pepMHC: Major histocompatibility complex binding peptide, PTX: paclitaxel, TRAIL: TNF-related apoptosis-inducing ligand.

**Table 2:** Merits and demerits, and research opportunities of three categories of recent advances in NP-based delivery system for cancer immunotherapy

Category	Summary	Merits	Demerits or research opportunities
ICD-inducing cytotoxic NPs	- Additional synergic mechanisms by ICD-inducing NPs may be used in current cancer immunotherapy to improve therapeutic efficacy - Cytotoxic NPs modify immunosuppressive conditions in tumors by facilitating immunogenic antigens and enhancing ICD to potentiate cancer immunotherapy	- Maximal therapeutic efficacy with low toxicity - Available with conventional cytotoxic agents - Established in many preclinical and clinical tests - They could potentiate other cancer immunotherapies - A number of clinically available cytotoxic drugs for ICD	- Risk of cytotoxic drug-induced systemic toxicity - Unpredictable therapeutic efficacy of cytotoxic drugs - Need for further clinical studies - Potential need for adjuvant or immune-stimulating agents
Cytokines and cytokine-like immune modulators NPs	- Delivery of cytokine and cytokine-like immune modulators to target cells using NPs can overcome short half-lives and low stability in blood- NPs selectively trigger cancer cell apoptosis and elicit immune response in leukocytes - Multifaceted and Janus NPs provide simultaneous targeting of tumor and immune cells to boost anticancer activity	- Delivery cytokines to target sites with minimal adverse and off-target effects - Reduced cytokine or cytokine-like immune modulator degradation in blood- Easy surface presentation of various immune modulators - Enhanced anti-tumor immune response	- Further mechanistic and translational studies - Risk of cytokine or immune modulator resistance - Limited utilization in personalized immunotherapy
NPs for adjuvant delivery	- Co-delivery of adjuvant and antigen using NPs for effective immunotherapy - Adjuvant delivery with cancer antigens using NPs more effective in immune cells of LNs resulting in antigen cross-presentation	- Effective co-loading and delivery of adjuvant and antigen to LNs - Various administration routes - Easy modulation of adjuvant biophysical properties	- Limited utilization in personalized immunotherapy - Unintended polarization may occur via adjuvant modification - Weak stability for large-scale manufacture

ICD: Immunogenic cell death, LNs: Lymph nodes, NPs: Nanoparticles

## Abbreviations

AlbiAg: albumin-binding antigen
AlbiCpG: albumin-binding adjuvant
AlbiVax: albumin-binding vaccines
Alum: aluminum salts
APCs: antigen-presenting cells
CpG ODNs: CpG oligodeoxynucleotides
CRT: cell surface-exposed calreticulin
EPR: enhanced permeability and retention
FDA: Food and Drug Administration
HA: hyaluronic acid
HES-D-IL-2: IL-2 cytokines conjugated on the surface of hydroxyethyl starch
HMGB1: high mobility groups protein B1
ICD: immunogenic cell death
IDO: indoleamine 2,3-dioxygenase
IL-2: interleukin-2
IND: indoximod
INF: interferon
LDH: layered double hydroxide
NLRP3: NOD-, LRR- and pyrin domain-containing protein 3
LNs: lymph nodes
LPS: lipopolysaccharide
MMP: matrix metalloproteinase
MSNPs: mesoporous silica nanoparticles
NP: nanoparticle
OVA: ovalbumin
OX: oxaliplatin
PD: pharmacodynamics
PD-L1: programmed cell death protein-ligand 1
PK: pharmacokinetics
poly I:C: polyinosinic:polycytidylic acid
PTX: paclitaxel
SVNPs: synthetic vaccine nanoparticles
Th1: T helper cell type 1
Th2: T helper cell type 2
TILs: tumor-infiltrating lymphocytes
TLR: toll-like receptor
TME: tumor microenvironment
TNF: tumor necrosis factor
TRAIL: tumor necrosis factor related apoptosis-inducing ligand
